# Accommodations provided and used to assess the cognitive performance of children with multiple disabilities resulting from Congenital Zika Syndrome

**DOI:** 10.1186/s41155-026-00391-4

**Published:** 2026-05-12

**Authors:** Zelma Freitas Soares, Andréa Perosa Saigh Jurdi, Cíntia Figueiredo Amaral, Leticia Marques dos Santos, Michelle Castro Montoya Flores, George Anderson Alves dos Santos, Vanessa Madaschi, Darci Neves dos Santos

**Affiliations:** 1 Instituto de Saúde Coletiva (ISC), Universidade Federal da Bahia (UFBA), Rua Basílio da Gama. s/n. Canela, Salvador/Ba, CEP 40110-040 Brazil; 2 Department of Health, Education and Society, Universidade Federal de São Paulo, Baixada Santista/SP, Brazil; 3 CEO VMCorporation, Rio de Janeiro/RJ, Brazil

**Keywords:** Bayley, Accommodations, Congenital Zika Syndrome, Cognitive performance, Children

## Abstract

**Background:**

The lack of development assessment instruments aimed at children with disabilities in the early years of life means that standardized items are used for accommodation.

**Objective:**

This study aimed to describe accommodations implemented in the administration of the Bayley Scales of Infant Development (BSID-III) cognitive scale and analyze their association with the cognitive performance of children affected by Congenital Zika Syndrome.

**Methods:**

A total of 125 children were assessed at 12 months using BSID-III. Twelve types of accommodation strategies were adopted and organized according to participants’ main disability conditions, such as sensory, motor, auditory, and general accommodations.

**Results:**

This study identified that 59.2% of participants used some accommodation. “Lighting” (sensory accommodation) included the use of lighting during the test, and “not timing” includes disabling time tracking for tasks that involve time management. They were the most used accommodations, in addition to having presented a statistically significant association (p < = 0.05) with several items assessed by the instrument. A strong, statistically significant negative correlation (ρ = -0.73; *p* < 0.001) was found between the number of accommodations used and the cognitive performance score obtained by children. This indicates that the greater the use of accommodations, the lower the level of cognitive performance. It is understood that the more severe the congenital syndrome, the more accommodations are required during the assessment.

**Conclusion:**

These findings are consistent with the guidelines provided in the accommodation procedures, which aim to offer opportunities for adequate assessment of children and allow for an explanation of their actual level of development, without any compensation. Assessment using accommodation provides more reliable data on children’s developmental strengths and weaknesses and has the potential to guide effective intervention programs. Such explanation favors a perspective of equity in the provision of care to children with disabilities.

## Introduction

The first years of life are a unique stage of development with the potential to predict developmental conditions in later stages. Consequently, investments in assessments to monitor development and identify strengths and weaknesses for early intervention implementation have become more common. However, current assessment instruments in the first years of life are commonly appropriate for children without severe impairments (Lamônica & Ferreira-Vasques, 2016; Lourenção & Bruzi, 2020). According to Law 13.146 (2015), a person with a disability is someone with a long-term impairment of a physical, mental, intellectual, or sensory nature resulting from congenital, hereditary, or acquired characteristics throughout life. Severe disability involves serious, long-lasting impairments that significantly limit a person’s ability to perform important activities of daily living and require substantial support (Wolfe & Tarnai, 2009). Disability is considered an experience of human vulnerability constructed in the interaction between body, environment, and social relations. A person with a disability, therefore, is understood as someone who encounters environmental barriers, rather than someone reduced to their limitation (Brasil, 2015; Ferreira et al. 2018; Organização Mundial da Saúde (OMS), 2003).

Although efforts have been made to develop instruments that consider specific disabilities, measuring the development of children with disabilities, especially severe disabilities, still faces considerable challenges (Mayes, 1999). Concomitantly, the use of accommodating items from previously standardized instruments in populations of typically developing children has become increasingly common in the assessment of children with disabilities (Ruiter et al., 2011; Visser et al., 2014).

Accommodation consists of minor changes to the test application instructions, stimulus materials, and response options for the items. However, the instrument’s intended measurement remains unchanged (Batshaw et al., 2002). In other words, accommodation involves changes only in instrument administration, and must preserve the construct validity of the original instrument version. Large-scale standardization research is not required to apply accommodations due to the possibility of using reference values from the instrument’s original standardization tables (Visser et al., 2014). However, accommodations should not be made indiscriminately. They should be guided by the objective of what is intended to be measured. Moreover, the accommodations used should be systematically recorded in a report.

The main objective of accommodations is to minimize the impact of a disability when administering instruments that assess children’s developmental skills (Ruiter et al., 2011). This allows children to be assessed accurately and have their actual level of development revealed. Therefore, accommodation aims to overcome obstacles that could prevent children with disabilities to be appropriately tested with the measure standardized. For instance, if a child has a hearing impairment, an accommodation may be used to augment their access to the measure’s auditory stimuli. This procedure is fundamental to ensuring that children with different types of disabilities can demonstrate their true abilities and reduce biases associated with limitations. Accommodation allows the assessment to more realistically capture children’s functional repertoire, increasing the validity and accuracy of the results by reducing the risk of underestimating abilities or overestimating deficits. Thus, assessments that use accommodations provide more reliable data on children’s developmental strengths and difficulties. This data is essential for implementing effective intervention programs because it informs the development of objectives, the selection of intervention strategies that align with the identified needs, and the monitoring of progress over time (Ruiter et al., 2011). Therefore, accommodation contributes to equity in the quality of care for children with disabilities (Campos et al., 2024; Souza et al., 2019).

Among the instruments designed to assess development throughout early childhood, the Bayley Scales of Infant and Toddler Development (BSID-III) (Bayley, 2006) offers accommodations within the manual itself. These accommodations are considered a way to increase the validity of inferences made by providing an opportunity to demonstrate real performance in a given area of development and by reducing the impact of deficiencies on what is being measured (Bayley, 2006). First published in 1969, the BSID-III is considered the gold standard for assessing child development. They provide a comprehensive assessment of different dimensions, including cognition, language, motor skills, and socio-emotional skills, and consistently demonstrate validity and reliability (Cruz et al., 2022; Madaschi et al., 2016). The BSID-III was adopted as an assessment instrument for the research project encompassing the study sample, “Effects of Congenital Neurological Manifestations Associated with Zika Virus on Child Development: A Prospective Cohort Study in the Context of Primary Care in Salvador, BA” (Santos et al., 2022).

Congenital Zika Syndrome (CZS) emerged in Brazil in 2015 as a serious public health crisis. CZS is caused by Zika virus infection during pregnancy, particularly in the first trimester. This infection results in structural and functional alterations in the fetus that have developmental consequences throughout life. The aforementioned project assessed the developmental consequences experienced by children born during the Zika virus epidemic. The researchers prioritized the home and Primary Health Care Units as settings for administering the assessment procedure (Santos et al., 2022). This ecological assessment, conducted within the context of children’s lives, revealed the various ways in which Zika virus infection can affect child development. Moreover, the Bayley Scale offers several ways to facilitate the assessment of children with disabilities. These include the presentation of items and materials, response time, and format, as well as feedback (Bayley, 2006).

Assessment tools that accommodate multiple disabilities, such as CZS (França et al., 2016, 2018), provide more accurate ability estimates than instruments with conventional application procedures. Although there are few studies on BSID-III accommodations for children with CZS, there are international studies on accommodations for other disabilities. A study that adapted the Motor Scale to assess children with motor disabilities found that those with disabilities had significantly higher scores on the accommodated version of the scale compared to the original version (Ruiter et al., 2010). In contrast, the scores of typically developing children did not differ between the original and accommodated versions of the scale, supporting the assumption that the item content and difficulty remained unchanged in the accommodated version. A second study (Visser et al., 2013) that included children with motor disabilities found that accommodations improved the scores of children with disabilities but had no effect on the scores of typically developing children. Additionally, a study examining the impact of accommodations for children with low vision revealed no statistically significant differences in children’s scores between the original and accommodated versions (Ruiter et al., 2011). Furthermore, another study that included children with visual and motor impairments found significant differences in scores between the accommodated and original versions only among children with motor impairments (Visser et al., 2014).

A systematic review (Cruz et al., 2022) of Brazilian literature on the BSID-III, which included 24 studies (12 of which were conducted with children with Down syndrome, twin-to-twin transfusion syndrome, or cystic fibrosis), revealed significant developmental delays in the assessed children. However, the studies did not include accommodations in the items of the instruments, nor did they carry out a comprehensive assessment. Instead, they prioritized only one or two domains (e.g., language and motor) and were predominantly carried out in outpatient and inpatient contexts. No assessments were carried out in the ecological context of children’s homes.

Furthermore, existing studies on the use of accommodations (Ruiter et al., 2011/2012; Visser et al., 2013/2014) were conducted in clinical settings with small samples and did not include an assessment of the cognitive domain, both nationally and internationally. Cognition is directly related to how a child understands the world, learns, communicates, solves problems, and participates in social and school activities (Cruz et al., 2022). It is fundamental to develop strategies that enable the assessment of children’s cognitive domain. This population-based study aimed to describe accommodations implemented in the administration of the BSID-III cognitive scale and analyze their association with the cognitive performance of children affected by CZS.

Two questions guided this investigation: (1) Is there a statistically significant association between the use of accommodation and the child’s score on BSID-III items; (2) How are the number of accommodations used correlates to the cognitive score obtained by the child?

## Methods

### Study design

This study involves developing and applying an accommodation protocol to measure the cognitive performance of children with multiple disabilities in a CZS-affected population. This is a cross-sectional study, with data linked to the DICa-Salvador cohort baseline (Santos et al., 2022).

### Study participants

A total of 125 children born between August 1, 2015, and July 31, 2016, in Salvador/BA, with a confirmed imaging diagnosis of CZS, participated in the study, according to procedures adopted by the Strategic Information Center for Health Surveillance of the City of Salvador (In Portuguese, *Centro de Informações Estratégicas de Vigilância em Saúde da Prefeitura Municipal de Salvador* - CIEVS-SMS).

### Sample selection

Of the 224 children originally diagnosed with CZS, 59 were not recruited for the cohort study for various reasons: addresses not located (19); refusals (23); change of municipality (15); and deaths (2). Subsequently, another 18 children were excluded due to positive results for other congenital infectious diseases (i.e., syphilis, toxoplasmosis, rubella, cytomegalovirus infection, and herpes simplex). This left 147 participants. Of the total number of participants, 22 were considered losses due to failure to complete the accommodation use form during the assessment, called the “Instrument for Assessment with Accommodations”. This left 125 participants for analysis.

### Instruments

#### Bayley Scales of Infant and Toddler Development (BSID-III version)

Administered individually, this scale assesses overall infant development between 0 and 42 months (Bayley, 2006). The Bayley scale is composed of three subscales that assess three specific and independent areas: cognition, language, and motor skills. This study only analyzes cognitive performance assessed at 12 months of age. The cognitive subscale consists of 91 items that assess perceptual-motor, classification, and discrimination skills, as well as memory, numerical reasoning, habituation, problem-solving, and generalization. These skills provide insight into how children think, react, and learn about the world around them. The cognitive scale is applied in a playful manner using specific materials, such as various toys, blocks, and figures. The application lasts between 40 and 90 min. Bayley provides an objective, standardized measure that ensures comparability. It has therefore been widely applied in research assessing child development, determining differences between groups, defining risk groups, and planning interventions. This has been done both internationally (Yeh et al., 2018) and nationally (Cruz et al., 2022; Eickmann et al., 2016; Madaschi et al., 2016). The research has involved children with typical development (Cruz et al., 2022) as well as children with developmental impairments (Yeh et al., 2018), including those with congenital neurological disorders associated with the Zika virus (Wheeler et al., 2020).

#### Technical Manual of Accommodations for BSID-III

This procedure was necessary because most of participants in this study had multiple physical and sensory disabilities, with frequent movement restrictions and disabilities in sensory processing and integration. These aspects compromised their ability to recognize objects and interact with the activities and stimuli present in the cognitive scale items. Thus, accommodations were necessary during the assessment. The accommodations implemented by the research team were based on the BSID-III manual’s (Bayley, 2006) recommendations, as well as on the foundations of the theory of development of children with multiple disabilities. Developmental theories consistently recognize the interdependence between developmental domains. For instance, impairments in sensory and motor skills, particularly fine motor skills, can directly affect cognitive abilities (Santos et al., 2013). Similarly, cognitive delays are associated with delays in language and communication (Viana et al., 2014). Development can occur even if it does not follow normative patterns. In this context, neuronal plasticity is influenced by the quality of interactions and early intervention, particularly when implemented during the first three years of life (Inguaggiato et al., 2017).

Therefore, a manual was prepared with accommodations for the BSID-III, instrument that adopts normative references based on a North American population (Bayley, 2006). The manual with accommodations is organized into sections according to the main types of disabilities found among the children who participated in the study. The sections include accommodations for children with visual impairments, accommodations for children with motor impairments, accommodations for children with hearing impairments, and general accommodations (Araújo et al., 2020). There are a total of 12 accommodations. Sensory accommodations include lighting control, image magnification, use of contrast, and object handling. Motor accommodations were defined as adjustments to the positioning of patients lying down, sitting on the mat, and on the lap, including manual facilitation to inhibit thumb insertion. General accommodations were defined as test task division, use of sign language, absence of stopwatch for the test, attempt scoring, and assistance in object maintenance. Further details about accommodation procedures can be found directly in the “Sensory and Motor Facilitation Protocol Manual for Use of the Bayley Scale of Infant Development – ​​BSIDIII in Children with Congenital Zika Syndrome” (Araújo et al., 2020) and in the supplementary file.

The team of researchers responsible for collecting the data received extensive training in the theoretical aspects of child development, as well as in how to administer the BSID-III with accommodations.

#### Accommodation record form

This form was used to record the use of accommodations adopted during assessment performed using the BSID-III. The manual described all 12 accommodations, which were listed in the instrument for assessment. Researchers marked with an X the accommodations used with each participant for each item on the cognitive scale. Two properly trained independent evaluators carried out an assessment. The inter-evaluator reliability, as measured by Cohen’s Kappa coefficient, was 0.92 (Silva et al., 2024). This value is considered excellent.

### Data collection procedures

Data collection took place at home between April 2017 and March 2018. Occasionally, applications took place at Primary Health Care Units when access to the family home was difficult. During the initial visit, guardians were informed about the study and signed an Informed Consent Form to proceed with the assessment. Two properly trained evaluators, psychologists and graduate students, carried out the assessment in a standardized manner (Silva et al., 2024). Minimal preparation was made to the environment for the test, which included the use of mats and a small table to present some tasks. While one evaluator administered the test and recorded the results using the original BSID-III materials, the second evaluator assisted with handling the children and organizing materials. When children had difficulty performing or were unable to perform the tasks provided in each item of the cognitive scale, the examiner used accommodations and recorded each one on the “Accommodation Record Form”, which was attached to the BSID-III application results sheet (Araújo et al., 2020). In other words, the researchers tested the children without accommodations. If a child did not score on an item, an accommodation was offered. The process of applying the research instruments took family routines into account, respecting mealtimes, bath times, and sleep times. Priority was given to times when the children were alert and not wearing restrictive clothing.

### Variable definition

A total of 12 accommodations, as defined in the BSID-III Technical Manual of Accommodations (Araújo et al., 2020), were considered as variables. They were all categorized dichotomously as 0 = not used and 1 = used. The accommodations were organized into four categories according to the number in which they were used in the cognitive assessment of each child: between 1 and 3; between 4 and 6; between 7 and 9; and between 10 and 12.

Items/tests from the cognitive scale that were accommodated were also defined as variables considering the skills assessed by each. Each accommodated item on the cognitive scale was categorized as 0 (when the accommodated item’s task was not scored) or 1 (when the accommodated item’s task was scored).

Cognitive performance was analyzed using the composite score of the cognitive scale, which varies between 40 and 160 points, with an average of 100 points (SD = 15) serving as the reference. A qualitative classification according to BSID-III (Bayley, 2006) was used for categorization. This classification has seven categories: high superior (score ≥ 130); superior (120–129); high average (110–119); average (90–109); low average (80–89); borderline (70–79); and extremely low (≤ 69).

### Analysis plan

Descriptive statistics, including absolute frequencies and percentages, were used to describe the accommodation variables and cognitive scale items. Fisher’s exact test was performed to investigate the association between the use of a specific accommodation and successfully executing (scoring) the corresponding cognitive items. This test was specifically chosen over the chi-square test to ensure accuracy in the presence of low expected frequencies in the 2 × 2 contingency tables. For all statistically significant associations, the Odds Ratio (OR) and its 95% Confidence Interval were calculated to quantify the strength and direction of the relationship.

Spearman’s rank correlation coefficient (p) was used to assess the relationship between the total number of accommodations utilized and the overall cognitive development scores. According to standard benchmarks, the magnitude of the correlation was interpreted as follows: coefficients between 0.10 and 0.39 were considered weak; between 0.40 and 0.69, moderate; and 0.70 or above, strong. All statistical procedures were conducted using Stata 12.0, maintaining a significance level of *p* = 0.05 for all tests.

Absolute frequencies and percentages were calculated for the accommodation variables and the cognitive scale items. Fisher’s exact test and Spearman’s coefficient were used, respectively, as alternatives to the chi-square test and Pearson’s coefficient to verify the relationship between accommodations and accommodated items and the correlation between the number of accommodations and cognitive performance, depending on the nonparametric distribution of the sample. These analyses were performed using IBM® Stata 12.0.

### Ethical aspects

This study rigorously complied with all items and requirements of the Brazilian National Council on Ethics in Human Research, in accordance with Brazilian National Health Council (In Portuguese, *Conselho Nacional de Saúde* - CNS) Regulations 466/2012 and 510/2016. The Research Ethics Committee of the Institute of Collective Health at the *Universidade Federal da Bahia* approved the original research project that provided the data under Opinion 1,659,107.

## Results

A total of 125 children participated in the study, 41.6% (*n* = 52) of whom were male. At the time of assessment, children’s ages ranged from 410 to 876 days old, with an average age of 610 days (SD = 70.8). Most caregivers were mothers (90.4%; *n* = 113). The sociodemographic profile revealed the following: 86.4% (*n* = 108) identified as black or pardo (mixed-race); 83.2% (*n* = 104) were unemployed; and 57.6% (*n* = 72) reported a monthly income equal to or less than one minimum wage.

Of the 125 children who participated, 59.2% (74) used some of the accommodations provided for cognitive performance assessments. Regarding the frequency with which accommodations were used, it was found that 44.61% of children used one to three accommodations, 16.25% used four to six accommodations, 31.06% used seven to nine accommodations, and 8.15% used 10 to 12 accommodations.

The following sensory accommodations were among the most frequently used (Table 1): “use of lighting in the test” (35.2%); “use of yellow contrast in the test” (26.4%); and “permission to handle the object” (28.8%). Among motor accommodations, “manual facilitation” was the most common (27.2%), while “not timing the test” was the most common (42.4%) among procedural accommodations.

**Table 1:** Frequency of sensory, motor, and general accommodations used in cognitive assessment of children affected by Congenital Zika Syndrome

Accommodations	*N* (125)	%
Sensory		
Use of lighting in the test	44	35.2
Use of yellow contrast in the test	33	26.4
Permission to handle the object	36	28.8
Motor		
Manual facilitation	34	27.2
Procedural (general)		
Not timing the test	53	42.4

Table 2 shows the frequency of the scores obtained for each assessed item according to the sensory accommodation procedure used. Regarding the “lighting” accommodation, all children (100%) scored on item 3 (observes the object for 3 s). For the remaining items in this category, the success rates were 76.9% for item 8 (observes the object for 5 s), 67.9% for item 12 (is used to the object), 36.7% for item 11 (demonstrates visual preference), and 25% for item 13 (prefers new objects). Statistical analysis using Fisher’s exact test revealed significant associations (*p* < 0.05) for items 8, 11, and 13. In all instances, the association was negative (OR <1.0), suggesting that the need for lighting accommodations indicates greater functional difficulty. These children were significantly less likely to score compared to those who did not require the aid. Item 12 did not reach statistical significance (*p* = 0.06), though it followed a similar negative trend (OR = 0.39).

**Table 2:** Score frequencies obtained for each item assessed according to the type of accommodation used

Accommodation / Item	Total *N*	Scored *n* (%)	OR (95% CI)	*p*-value	Association
Lighting accommodation					
Item 3 (observes object for 3 s)	27	27 (100.0)	--	1.00	Non-significant
Item 8 (observes object for 5 s)	26	20 (76.9)	0.11 [0.02; 0.55]	< 0.01	Negative
Item 11 (shows visual preference)	30	11 (36.7)	0.25 [0.09; 0.66]	< 0.01	Negative
Item 12 (gets used to the object)	28	19 (67.9)	0.39 [0.13; 1.17]	0.06	Negative
Item 13 (prefers new objects)	24	6 (25.0)	0.15 [0.04; 0.44]	< 0.01	Negative
Yellow contrast accommodation					
Item 3 (observes the object for 3 s)	10	10 (100.0)	--	1.00	Non-significant
Item 8 (observes the object for 5 s)	13	10 (76.9)	0.19 [0.13; 1.37]	0.06	Non-significant
Object handling accommodation					
Item 16 (explores the object)	13	6 (46.2)	0.52 [0.13; 1.93]	0.37	Non-significant
Item 17 (takes the object to the mouth)	11	7 (63.6.8)	1.02 [0.24; 5.04]	1.00	Non-significant
Manual facilitation accommodation					
Item 16 (explores the object)	21	9 (42.9)	0.42 [0.14; 1.19]	0.09	Non-significant
Item 17 (takes the object to the mouth)	15	7 (46.7)	0.46 [0.13; 1.59]	0.17	Non-significant
Not timing accommodation					
Item 11 (shows visual preference)	32	11 (34.4)	0.18 [0.07; 0.44]	< 0.01	Negative
Item 12 (is used to the object)	26	17 (65.4)	0.34 [0.12; 1.03]	0.047	Conflict
Item 13 (prefers new objects)	31	12 (38.7)	0.30 [0.12; 0.74]	< 0.01	Negative
Item 14 (is used to the illustration)	26	7 (26.9)	0.08 [0.02; 0.23]	< 0.01	Negative
Item 15 (prefers new illustration)	32	1 (3.1)	0.02 [0.00; 0.12]	< 0.01	Negative

*Fisher’s exact test (p-value <= 0.05)

Among the children who utilized the “yellow contrast” accommodation, 100% scored on item 3 (observes the object for 3 s), while 76.9% scored on item 8 (observes the object for 5 s). Fisher’s exact test revealed no statistically significant association between these items and the use of this accommodation (*p* = 1.00 and *p* = 0.06, respectively). This indicates that using this accommodation did not significantly alter the probability of success compared to not using it.

As for the “object handling” accommodation, success rates were 46.2% for item 16 (explores the object) and 63.6% for item 17 (takes the object to the mouth). Similarly, Fisher’s exact test revealed no statistically significant association (*p* = 0.37 and *p* = 1.00, respectively), suggesting that performance on these manual tasks was independent of this specific accommodation procedure.

In relation to the children who used the “manual facilitation” accommodation, the success rates were 42.9% for item 16 (explores the object) and 46.7% for item 17 (takes the object to the mouth). Fisher’s exact test revealed no statistically significant association between these items (*p* = 0.09 and *p* = 0.17, respectively), suggesting that performance remained independent of the use of this accommodation.

Finally, for the “not timing” accommodation, scoring frequencies were 65.4% for item 12 (is used to the object), 38.7% for item 13 (prefers new objects), 34.4% for item 11 (shows visual preference), 26.9% for item 14 (is used to the illustration), and 3.1% for item 15 (prefers new illustration). Fisher’s exact test revealed a statistically significant association (*p* < 0.05) for all items in this category.

OR analysis revealed a negative association for items 11, 13, 14, and 15 (OR < 1.0). This indicates that the need for extra time is a marker of greater functional difficulty. However, item 12 presented a unique statistical conflict. Although it was significant (*p* = 0.047), its confidence interval (0.12–1.03) included the null value. This suggests that the association is borderline or unstable.

In relation to the cognitive performance levels of children who used accommodations, 98.65% had composite scores classified as extremely low (≤69), while only 1.35% were classified as borderline (70-79). Conversely, 47.1% of children who did not use accommodations had average (90–109) composite cognitive performance scores, while 23.5% had extremely low scores (≤ 69) (Table 3).

**Table 3:** Cognitive performance among children affected by Congenital Zika Syndrome according to the use of accommodations in the Cognitive Scale application

Cognitive performance	Used accommodation (*N*=74)		Did not use accommodation (*N*=51)	
	*N*	%	*N*	%
Extremely low	73	98.7	12	23.5
Borderline	1	1.3	2	3.9
Low average	_	_	6	11.8
Average	_	_	24	47.1
High average	_	_	5	9.8
Superior	_	_	2	3.9
High superior	_	_	_	_

Composite score classified by the following categories: High superior = score ≥ 130; Superior = 120-129; High average = 110-11; Average = 90-109; Low average = 80-89; Borderline = 70-79; and Extremely low = ≤ 69

Using Spearman’s coefficient, we observed a strong and statistically significant negative correlation (ρ = -0.73; *p* < 0.001) between the number of accommodations used and the cognitive performance score obtained when investigating correlations between the number of accommodations and children’s cognitive performance scores. This indicates that the greater the use of accommodations, the lower the level of cognitive performance of children affected by CZS.

## Discussion

This study aimed to describe accommodations implemented in the administration of the BSID-III cognitive scale and analyze their association with the cognitive performance of children affected by CZS who would not have been assessed using conventional methods. Of the 125 children with CZS who participated in this study, 59.2% used at least one of the 12 accommodations listed in the “BSID-III Technical Manual of Accommodations”. The most frequent use (44.61%) was up to three accommodations per child.

The “lighting” and “no timing” accommodations were the most commonly used and were associated with several items assessed, as determined by Fisher’s exact test. These results suggest that not all accommodations were equally effective in minimizing the impact of multiple disabilities on cognitive assessment test performance. Sensory accommodations related to the way materials or objects were presented and not timing also had an important performance in the assessment of children with disabilities in previous studies that performed accommodations with the BSID-III (Ruiter et al., 2011; Visser et al., 2013). The frequent use of sensory accommodations in the cognitive assessment of children participating in the present study is possibly justified by the fact that children with neurological alterations resulting from CZS tend to have low vision and altered sensitivity to contrasts. These children may have difficulty perceiving objects and facial expressions. They also have difficulty adapting to lighting, which may be due to neurological irritation in the central nervous system (Ventura et al., 2017). Furthermore, children with multiple disabilities tend to have slower sensorimotor processing when performing tasks and activities (Visser et al., 2013/2014), which possibly explains the recurring use of “not timing” accommodation in the cognitive assessment of the children participating in this study.

Although associations were found between the aforementioned accommodations (“lighting” and “not timing”) and the various items in the instrument, children who used accommodations more frequently had composite scores classified as “extremely low performance” compared to children who did not use accommodations. Furthermore, the results of the correlation analysis revealed that the more accommodations children used, the lower their cognitive performance level. These findings may be due to the severity of multiple disabilities resulting from CZS (Marques et al., 2019; Moreira et al., 2018; Carvalho et al., 2019). It is reasonable to assume that the greater the impairment caused by the syndrome, the more accommodations that would be required for assessment. Some authors (Ruiter et al., 2010; Visser et al., 2013) emphasize the importance of exercising caution when using accommodations to ensure that this procedure does not lead to an overestimation of the performance scores of children with multiple disabilities. Thus, the data obtained in the present study reflect compliance with the objectives set forth in the literature on accommodations. These objectives consist of minimizing the impact of a given disability on developmental skills, providing children with the opportunity to be adequately assessed and have their actual level of development revealed without compensation (Ruiter et al., 2010/2011).

Unlike previous studies involving accommodations (Visser et al., 2013/2014), this study included children with multiple impairments due to CZS in the sample. While the degree of disability can predict developmental outcomes, its broad spectrum of manifestation underscores the importance of conducting refined assessments that can generate information to direct the implementation of stimulation programs sensitive to each child’s real developmental needs. Consistently assessing children with multiple disabilities is essential to providing them with stimulation resources and promoting their inclusion in school and other contexts. This ensures their status as subjects of rights. The Zika epidemic provided an opportunity to study the feasibility of accommodation procedures for children with microcephaly by capturing a large sample size. In contrast, previous studies on accommodation had small sample sizes and were conducted with children who had other disabilities, such as motor disabilities and low vision (Ruiter et al., 2010/2011; Visser et al., 2013/2014).

Among the limitations of this study, we noted that the non-accommodated version of the scale was not applied to the same group of children for comparison with the scores generated by the accommodated version. This made it difficult to determine precisely how the adaptations improved the performance of children with disabilities on the test. In any case, the decision to not subject children with CZS to two exhaustive assessment processes was made considering the previous literature on accommodations, despite having methodologically weakened the present study. Previous studies have shown differences in scores between the original and accommodated versions among children with disabilities. However, this result was not found when comparing the scores of children with typical development (Ruiter et al., 2010; Visser et al., 2013). In any case, future studies are needed to test whether the psychometric qualities of validity and reliability (Cunha et al., 2016) are indeed guaranteed in studies using accommodations.

Another limitation is the sample size. Although it is larger than those in previous studies on accommodations (Ruiter et al., 2010/2011; Visser et al., 2013/2014), it is still considered small for generalizing the findings. Furthermore, although the BSID-III is considered the gold standard for assessing child development (Cruz et al., 2022; Madaschi et al., 2016), caution is needed when interpreting its results in this study. Although efforts have been made toward cross-cultural adaptation (Madaschi et al., 2016), the adopted normative reference is based on a North American population (Bayley, 2006). The development of a psychometric instrument is based on the cultural and relational characteristics of the local population. In general, the North American context features parenting practices that value the early development of children’s autonomy, their individual exploration of objects, and direct interaction between adults and children (Gomide & Otoni da Silva, 2025). These aspects are strongly reflected in the items of the BSID-III (Bayley, 2006). In the Brazilian context, particularly among socioeconomically vulnerable families, which accounted for the largest number of Zika virus-affected children (Souza, Natividade, Werneck, & Santos, 2022), parenting practices are often characterized by family interdependence, multiple caregivers, and a lack of emphasis on highly structured toys (Gomide, 2022). To some extent, these differences may influence a child’s performance on tasks that require familiarity with specific materials or expected responses in formal testing contexts. In any case, it is noteworthy that this is the first Brazilian study to use accommodations in BSID-III items (Cruz et al., 2022). Its results reveal specifics that could guide future research, particularly that involving children with multiple disabilities resulting from CZS.

## Conclusion

This study describes and analyzes the accommodations used in the cognitive assessment of children affected by CZS. The findings dialogued with the guidelines provided in the accommodation procedures regarding their function of offering opportunities for an adequate assessment of children with disabilities. These changes occurred in the application instructions, stimulus materials, and response possibilities for the test items, but there was no compensation. This allows a child’s actual developmental level to be demonstrated and promotes equity in the provision of care to children with disabilities.

## Data Availability

Due to the sensitive nature of the information collected in this study, guardians the children participants were assured raw data would remain confidential and would not be shared.

## Abbreviations

BSID-III: Bayley Scales of Infant and Toddler Development
CZS: Congenital Zika Syndrome
CIEVS: Estratégicas de Vigilância em Saúde
SMS: Secretaria Municipal de Saúde

## References

[CR1] Araújo, C. F., Cabral, C. B., Dantas, J., Oliveira, K. N. R., Flores, M. C. M., Almeida, T. M., Silva, T. C. L., Santos, L. M., & Santos, D. N. (2020). Manual do protocolo de facilitações sensoriais e motoras para uso da escala Bayley de desenvolvimento infantil – BSIDIII em crianças com síndrome congênita do zika vírus. http://repositorio.ufba.br/ri/handle/ri/31933

[CR2] Batshaw, C. E., Church, R. P., & Batshaw, M. L. (2002). Special education services. In M. L. Batshaw (Ed.), Children with disabilities. 6th ed. Baltimore: Paul H. Brookes Publishing Co. 2007:523-538. http://search.ebscohost.com/login.aspx?direct=true&db=psyh&AN=2007-03652-034&site=ehost-live&scope

[CR3] Bayley, N. (2006). *Manual of Bayley Scales of Infant DevelopmentTM – Third Edition*. The Psychological Corporation. 10.1037/t14978-000

[CR4] Brasil (2015). *Lei nº 13.146, de 6 de julho de 2015*. Institui a Lei Brasileira de Inclusão da Pessoa com Deficiência (Estatuto da Pessoa com Deficiência). Diário Oficial da União: seção 1, Brasília, DF. http://www.planalto.gov.br/ccivil_03/_ato2015-2018/2015/lei/l13146.htm

[CR5] Campos, R. B., Alderete, G., Silva, R. M. M., & Zilly, A. (2024). Itinerário terapêutico de crianças com necessidades especiais de saúde: revisão integrativa. *Rev Eletr Enferm*, *26*(77290), 1–11. 10.5216/ree.v25.77290

[CR6] Carvalho, A., Brites, C., Mochida, G., Ventura, P., Fernandes, A., Lage, M. L., Taguchi, T., Brandi, I., Silva, A., Franceschi, G., Lucena, P., & Lucena, R. (2019). Clinical and neurodevelopmental features in children with cerebral palsy and probable congenital Zika. *Brain & development*, *41*(7), 587–594. 10.1016/j.braindev.2019.03.005 30914212 10.1016/j.braindev.2019.03.005

[CR7] Cruz, E. J. S., Lima, S. S., Cavalcante, L. I. C., & Pedroso, J. S. (2022). Uso da Escala de Avaliação do Desenvolvimento Infantil Bayley III em Crianças Brasileiras: Revisão Sistemática. *Psicologia: Teoria e Pesquisa*, *38*, e38. 10.1590/0102.3772e38320.pt

[CR8] Cunha, C. M., Almeida-Neto, O. P., & Stackfleth, R. (2016). Principais métodos de avaliação psicométrica da validade de instrumentos de medida. *Rev Aten Saúde*, 75–83. 10.13037/rbcs.vol14n47.3391. São Caetano do Sul, v. 14, n. 47.

[CR9] Eickmann, S. H., Emond, A. M., & Lima, M. (2016). Avaliação do desenvolvimento infantil: além do neuromotor. *J Pediatr (Rio J)*, *92*, S71–S83. 10.1016/j.jped.2016.01.007 27012923 10.1016/j.jped.2016.01.007

[CR10] Ferreira, H. N. C., Schiariti, V., Regalado, I. C. R., Sousa, K. G., Pereira, S. A., Fechine, C. P. N. D. S., & Longo, E. (2018). Functioning and Disability Profile of Children with Microcephaly Associated with Congenital Zika Virus Infection. *International journal of environmental research and public health*, *15*(6), 1107. 10.3390/ijerph15061107 10.3390/ijerph15061107 PMC6025082 29844290

[CR11] França, G. V. A., Pedi, V. D., Garcia, M. H. O., Carmo, G. M. I., Leal, M. B., & Garcia, L. P. (2016). Síndrome congênita vírus Zika no Brasil: uma série de casos dos primeiros 1501 nascidos vivos com investigação completa. *The Lancet*, *388*(10047), 891–897.

[CR12] França, G. V. A., Pedi, V. D., Garcia, M. H. O., Carmo, G. M. I., Leal. M. B., & Garcia, L. P. (2018). Congenital syndrome associated with Zika virus infection among live births in Brazil: a description of the distribution of reported and confirmed cases in 2015-2016. *Epidemiol. Serv. Saúde*, Brasília, 27(2):e2017473. 10.5123/S1679-49742018000200014 10.5123/S1679-49742018000200014 29972474

[CR14] Gomide, P. I. C. (2022). Instrumentos nacionais de práticas parentais: Uma revisão sistemática da literatura. *Psicologia Argumento*, *40*(109), 1776–1796. 10.7213/psicolargum.40.109.AO06

[CR13] Gomide, P. I. C., & da Otoni, F. (2025). Instrumentos internacionais de práticas parentais utilizados no Brasil: Uma revisão de literatura. *Interação em Psicologia*, *28*(3). https://revistas.ufpr.br/psicologia/article/view/86571

[CR15] Inguaggiato, E., Sgandurra, G., & Cioni, G., G (2017). Brain plasticity and early development: Implications for early intervention in neurodevelopmental disorders. *International Journal of Developmental Disabilities*, *63*(3), 133–135. 10.1080/20473869.2017.1325143

[CR16] Lamônica, D. A. C., & Ferreira-Vasques, A. T. (2016). Escalas de Desenvolvimento para Avaliação de Crianças. In: Giacheti, C. M. (org.). Avaliação da fala e da linguagem: perspectivas interdisciplinares. Marília: Oficina Universitária; São Paulo: Cultura Acadêmica. pp. 193-208. 10.36311/2016.978-85-7983-782-1.p193-208

[CR17] Lourenção, L. F. P., & Bruzi, F. A. F. (2020). Aplicação e utilização do teste Denver II na avaliação do desenvolvimento infantil: uma revisão de literatura. *Revista Saúde e Desenvolvimento v*, *14*, 17.

[CR18] Madaschi, V., et al. (2016). Bayley-III scales of infant and toddler development: Transcultural adaptation and psychometric properties. *Paideia*, *26*, 189–197. 10.1590/1982-43272664201606

[CR19] Marques, F. J. P., Teixeira, M. C. S., Barra, R. R., de Lima, F. M., Dias, B. L. S., Pupe, C., Nascimento, O. J. M., & Leyser, M. (2019). Children Born With Congenital Zika Syndrome Display Atypical Gross Motor Development and a Higher Risk for Cerebral Palsy. *Journal of child neurology*, *34*(2), 81–85. 10.1177/0883073818811234 30421639 10.1177/0883073818811234

[CR20] Mayes, S. D. (1999). Mayes Motor-Free Compilation (MMFC) for Assessing Mental Ability in Children with Physical Impairments. *International Journal of Disability Development and Education*, *46*(4), 475–485. 10.1080/103491299100452

[CR21] Moreira, M. E. L., Nielsen-Saines, K., Brasil, P., Kerin, T., Damasceno, L., Pone, M., Carvalho, L. M. A., Pone, S. M., Vasconcelos, Z., Ribeiro, I. P., Zin, A. A., Tsui, I., Adachi, K., Gaw, S. L., Halai, U. A., Salles, T. S., da Cunha, D. C., Bonaldo, M. C., Gabaglia, R., Guida, C., & Cherry, L., J. D (2018). Neurodevelopment in Infants Exposed to Zika Virus In Utero. *The New England journal of medicine*, *379*(24), 2377–2379. 10.1056/NEJMc1800098 30575464 10.1056/NEJMc1800098 PMC6478167

[CR22] Organização Mundial da Saúde. (2003). *CIF: Classificação Internacional de Funcionalidade, Incapacidade e Saúde*. Editora da Universidade de São Paulo (EDUSP).

[CR23] Ruiter, S. A. J., Nakken, H., van der Meulen, B. F., & Lunenborg, C. B. (2010). Low Motor Assessment: A Comparative Pilot Study with Young Children With and Without Motor Impairment. *Journal of Developmental and Physical Disabilities*, *22*(1), 33–46. 10.1007/s10882-009-9165-5 20157361 10.1007/s10882-009-9165-5 PMC2817083

[CR24] Ruiter, S., Nakken, H., Janssen, M., Van Der Meulen, B., & Looijestijn, P. (2011). Adaptive assessment of young children with visual impairment. *British Journal of Visual Impairment*, *29*(2), 93–112. 10.1177/0264619611402766

[CR26] Santos, M. M., Corsi, C., Marques, L. A., & Rocha, N. A. (2013). Comparison of motor and cognitive performance of children attending public and private day care centers. *Brazilian Journal of Physical Therapy*, *17*(6), 579–587. 10.1590/S1413-35552012005000126 24346293 10.1590/S1413-35552012005000126 PMC4207148

[CR25] Santos, D. N., de Araújo, T. M., Dos Santos, L. M., Kuper, H., Aquino, R., Silveira, D., Miranda, I. H., Pereira, S. S., M., & Werneck, G. L. (2022). The Salvador Primary Care Longitudinal Study of Child Development (CohortDICa) Following the Zika Epidemic: Study Protocol. *International journal of environmental research and public health*, *19*(5), 2514. 10.3390/ijerph19052514 35270212 10.3390/ijerph19052514 PMC8909628

[CR27] Silva, T. C. L., Santos, S. G. A. A., Madaschi, L. M., Jurdi, V. A. P. S., & Santos, D. N. (2024). Confiabilidade na Aplicação das Escalas Bayley: Avaliando Crianças Acometidas pelo Zika Vírus. *Psicologia: Teoria e Prática*, *26*(2). 10.5935/1980-6906/ePTPPA14444.en. ePTPPA14444. São Paulo, SP.

[CR28] Souza, M. J., Morais, A. C., Lima, I. S., Mascarenhas, J. S., Lima, M. M., & Amorim, R. C. (2019). Itinerário terapêutico das famílias de crianças com microcefalia. *Rev Baiana Enferm Dec*, *33*, e32966. 10.18471/rbe.v33.32966

[CR29] Souza, M.,, A. P, Natividade, S, M., da, Werneck, L, G., Santos, N, D., & dos (2022). Congenital Zika syndrome and living conditions in the largest city of northeastern Brazil. *Bmc Public Health*, *22*(1), 1231. 10.1186/s12889-022-13614-x 10.1186/s12889-022-13614-x PMC9208747 35725427

[CR30] Ventura, L. O., Ventura, C. V., Lawrence, L., van der Linden, V., van der Linden, A., Gois, A. L., Cavalcanti, M. M., Barros, E. A., Dias, N. C., Berrocal, A. M., & Miller, M. T. (2017). Visual impairment in children with congenital Zika syndrome. *Journal of AAPOS: the official publication of the American Association for Pediatric Ophthalmology and Strabismus*, *21*(4), 295–299e2. 10.1016/j.jaapos.2017.04.003 28450178 10.1016/j.jaapos.2017.04.003

[CR31] Viana, T. P., Andrade, I. S. N. D., & Lopes, A. N. M. (2014). Desenvolvimento cognitivo e linguagem em prematuros. *Audiologia - Pesquisa de Comunicação*, *19*(1), 1–6. 10.1590/S2317-64312014000100002

[CR32] Visser, L., Ruiter, S. A. J., Van der Meulen, B. F., Ruijssenaars, W. A. J. J. M., & Timmerman, M. E. (2013). Validity and suitability of the Bayley-III Low Motor/Vision version: A comparative study among young children with and without motor and/or visual impairments. *Research in Developmental Disabilities*, *34*(11), 3736–3745. 10.1016/j.ridd.2013.07.027 24025434 10.1016/j.ridd.2013.07.027

[CR33] Visser, L., Ruiter, S. A. J., van der Meulen, B. F., Ruijssenaars, W. A.,, M. J.J., & Timmerman, M. E. (2014). Accommodating the Bayley-III for Motor and/or Visual Impairment. *Pediatric Physical Therapy*, *26*(1), 57–67. 10.1097/pep.0000000000000004 10.1097/PEP.0000000000000004 24356320

[CR34] Wheeler, A. C., et al. (2020). Developmental Outcomes Among Young Children With Congenital Zika Syndrome in Brazil. *JAMA Netw Open*, *3*, e204096. 10.1001/jamanetworkopen.2020.4096 32369180 10.1001/jamanetworkopen.2020.4096 PMC7201309

[CR35] Wolfe, P. S., & Tarnai, S. B., S. B (2009). Defining severe disabilities: Implications for research and practice. *International Journal of Special Education (IJSE)*, *24*(2), 19–28.

[CR36] Yeh, H. R., Park, H. K., Kim, H. J., Ko, T. S., Won, H. S., Lee, M. Y., Shim, J. Y., & Yum, M. S. (2018). Neurodevelopmental outcomes in children with prenatally diagnosed corpus callosal abnormalities. *Brain Dev*. Sep;40(8):634-641. 10.1016/j.braindev.2018.04.012. Epub 2018 May 23. PMID: 29801921. 10.1016/j.braindev.2018.04.012 29801921

